# A Chelation Strategy for *In-situ* Constructing Surface Oxygen Vacancy on {001} Facets Exposed BiOBr Nanosheets

**DOI:** 10.1038/srep24918

**Published:** 2016-04-26

**Authors:** Xiao-jing Wang, Ying Zhao, Fa-tang Li, Li-jun Dou, Yu-pei Li, Jun Zhao, Ying-juan Hao

**Affiliations:** 1 College of Science, Hebei University of Science and Technology, Shijiazhuang 050018, China; 2 College of Science, Agricultural University of Hebei, BaoDing, 071001, China

## Abstract

Surface defect of nanomaterials is an important physical parameter which significantly influences their physical and chemical performances. In this work, high concentration of surface oxygen vancancies (SOVs) are successfully introduced on {001} facets exposed BiOBr nanosheets via a simple surface modification using polybasic carboxylic acids. The chelation interaction between carboxylic acid anions and Bi^3+^ results in the weakness of Bi-O bond of BiOBr. Afterwards, under visible-light irradiation, the oxygen atoms would absorb the photo-energy and then be released from the surface of BiOBr, leaving SOVs. The electron spin resonance (ESR), high-resolution transmission electron microscopy (HRTEM), and UV–vis diffuse reflectance spectra (DRS) measurements confirm the existence of SOVs. The SOVs can enhance the absorption in visible light region and improve the separation efficiency of photo-generated charges. Hence, the transformation rate of adsorbed O_2_ on the as-prepared BiOBr with SOVs to superoxide anion radicals (•O_2_^−^) and the photocatalytic activity are greatly enhanced. Based on the modification by several carboxylic acids and the photocatalytic results, we propose that carboxylic acids with natural bond orbital (NBO) electrostatic charges absolute values greater than 0.830 are effective in modifying BiOBr.

Semiconductor photocatalysis has attracted a wide attention for its potential in solving energy and environmental issues^1^,^2^,^3^,^4^. In a typical photocatalytic process, charge kinetics mainly comprises three key steps: the generation of charges under photoexcitation, transfer to the surface, and consumption for redox reactions on the surface, which drive the conversion from solar to chemical energy^5^. Improving the efficiency of each step is fundamental way to develop highly efficient photocatalysts^6^,^7^. Thus, during the past years, various strategies, such as control of facets^8^,^9^,^10^, making defects^11^,^12^, semiconductor combination^13^,^14^,^15^, doping^16^,^17^,^18^, deposition of noble metals^19^, have been developed for improving the performance of semiconductor photocatalysts. At present, researchers have shown that the activity of photocatalysts is related not only to the crystal size and morphology, but also to the separation and transfer efficiency of the charges^20^.

In recent years, two-dimensional layer-structured inorganic photocatalysts such as carbon-containing compounds, transition metal oxides and transition metal dichalcogenides, have received massive research interest due to their unique structures and promising properties^21^,^22^,^23^,^24^,^25^,^26^. As a new class of layered materials, BiOX (X=F, Cl, Br, and I) compounds exhibited their extensive photocatalytic applications in energy conversion and environmental remediation^27^,^28^,^29^,^30^,^31^. Bismuth oxybromide (BiOBr) is a member of this family, which has proven to be a promising photocatalyst^32^,^33^,^34^,^35^,^36^,^37^. Nevertheless, monoclinic phase BiOBr photocatalyst still suffers from several disadvantages, such as limited visible-light responsive region and insufficient efficiency of photo-generated charges.

As a typical defect, Oxygen vacancy can expand or enhance the photo-absorption and photocatalytic performance of some monoclinic phase photocatalysts. Recently, Li *et al*.^11^ have fabricated BiOBr nanosheets with oxygen vacancies via a hydrothermal-reduction route. However, bulk oxygen vacancies can also act as charge carrier traps where photo-induced electron-hole pairs recombine, resulting in the decrease of photoactivity^38^. Hence, if oxygen vacancies can be constructed and controlled just on the photocatalyst surface, the charge carriers’ recombination would be inhibited. Furthermore, surface oxygen vacancies (SOVs) with abundant localized electrons are of particular interest for the enhanced adsorption and activation of O_2_ to reactive oxygen species, which could further inhibit the recombination of photo-generated charge carries^39^,^40^,^41^,^42^. However, it still remains a great challenge to control the concentration and extent of SOVs.

BiOX has a two-dimensional layered structure, the [X–Bi–O–Bi–X] slices stacked together by the non-bonding (van der Waals) interaction through the X atoms along c-axis. The separation of photo-generated electrons and holes in BiOX mainly takes place along the (001) direction; thus in general the {001} facets-dominated BiOX would exhibit higher photocatalytic ability^10^,^11^,^28^,^29^,^33^,^43^. On the other hand, Bi–O bond on the {001} facets is of long length and low energy; therefore it is easy for O atoms on the surface to release into atmosphere, leaving SOVs^28^. For example, oxygen vacancies can be easily generated on the BiOCl {001} facets under UV light irradiation^43^.

Inspired by abovementioned discussion and results, we present a novel chelation strategy of controllable *in-situ* SOVs introduction on {001} facets-dominated BiOBr nanosheets in this research. It is known that polybasic carboxylic acids have strong chelation ability; hence we employ several polybasic carboxylic acids for coordination with Bi ions on the surface of BiOBr. As a result, the bond strength of Bi-O is weakened, leading to the removal of O atoms under visible light illumination and the formation of SOVs. After the introduction and confinement of SOVs in {001} facet-dominated BiOBr nanosheets, the photocatalytic activity and transformation rate of adsorbed O_2_ reduced to •O_2_^−^ radicals are significantly improved.

## Results and Discussion

**Figure 1:**
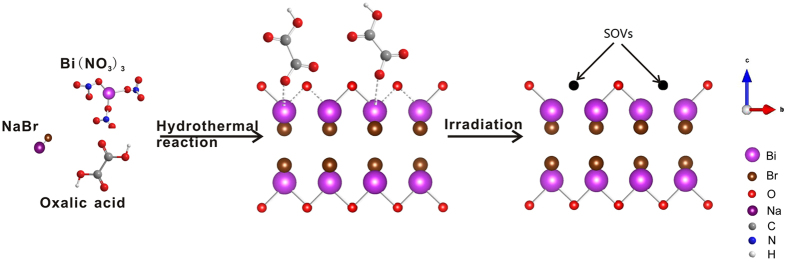
Schematic illustration of *in-situ* fabrication of surface oxygen vacancies.

*In-situ* fabrication of SOVs was realized via a hydrothermal strategy followed by irradiation treatment with oxalic acid as the modifier. The precursor for BiOBr was synthesized from bismuth nitrate. The covalent binding between Bi and O, and their highly dense arrangement in [Bi_2_O_2_] layers would strongly inhibit the diffusion of dopant precursor, resulting in a surface oxalic acid anions doping. Then irradiation treatment in liquid phase was performed to trigger the generation of SOVs. The potential formation process of SOVs is illustrated in Fig. 1.

**Figure 2:**
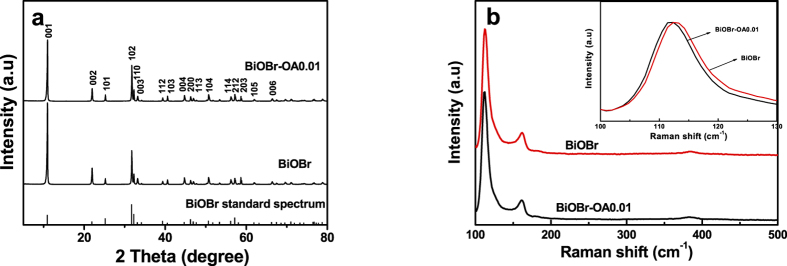
(**a**) XRD patterns and (**b**) Raman spectrum of the as-synthesized BiOBr and BiOBr-OA0.01 samples.

XRD patterns of bare BiOBr and oxalic acid modified BiOBr (BiOBr-OA0.01) samples are shown in Fig. 2a. Firstly, it can be seen that all of the as-prepared BiOBr samples have clear and intense diffraction peaks implying the good crystallinity of the samples. All the diffraction peaks are in good agreement with the tetragonal BiOBr phase (JCPDS NO. 01-078-0348), no obvious impurity peak obtained when the oxalic acid is used as the additive. Secondly, compared with the standard spectrum of BiOBr shown in the bottom of Fig. 2a, the as-prepared pristine BiOBr and BiOBr-OA0.01 have stronger relative peak intensity at (001) series planes, such as (002), (003) and (004), exhibiting the highly exposed {001} facets of BiOBr.

Raman spectrum was carried out to indentify the interaction between oxalic acid anions and BiOBr. Typically, Raman signals of pristine BiOBr have three kinds with A1g, B1g and Eg. The positions of the Raman signals are relative to the short-range order of BiOBr crystal. The three characteristic bands at 112.3, 161.8 and 385.2 cm^−1^ are assigned to the A1g internal Bi−Br stretching mode, the Eg internal Bi−Br stretching mode and the B1g bands generated by the motion of oxygen atoms, respectively^10^. As shown in Fig. 2b, in comparison with BiOBr, a red shift of the bands at 112.3 cm^−1^ in BiOBr-OA0.01 crystals was obtained, which indicates the corresponding structural change. This implies a feeble variation of lattice symmetry induced by the doping of oxalic acid.

**Figure 3:**
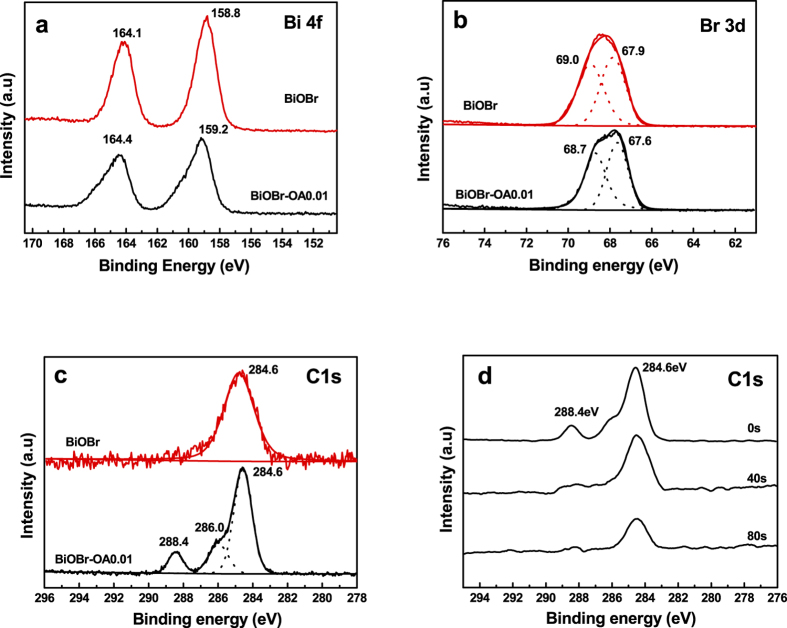
X-Ray photoelectron spectra of (**a**) Bi 4f, (**b**) Br 3d, (**c**) C1s and (**d**) Time-dependent high resolution C 1s XPS spectra upon Ar^+^ sputtering for BiOBr-OA0.01.

In order to further confirm the existence of oxalic acid anions and the interaction between oxalic acid anions and BiOBr, XPS analysis was performed. The high-resolution spectra of Bi 4f are shown in Fig. 3a. The two strong peaks centered at 158.8 and 164.1 eV for BiOBr are associated core lines of Bi 4f_5/2_ and 4f_7/2_. As for BiOBr-OA0.01, the Bi 4f_5/2_ and 4f_7/2_ peaks shift to 159.2 and 164.4 eV, respectively, indicating there are interactions between Bi and oxalic acid anions. The high-resolution spectra of Br 3d are shown in Fig. 3b. There are two peaks at 69.0 and 67.9 eV, which can be ascribed to Br 3d 5/2 and Br 3d, respectively^14^. However, the peaks in the BiOBr-OA0.01 sample are observed at 68.7 eV and 67.6 eV, which are lower than those for pure BiOBr. Such shifts can be attributed to the atomic interaction between Br^−^ and oxalic acid anions. Because of bilayer structure of BiOBr, some adsorbed oxalic acid anions can be reserved in the surface bilayer structure of [Bi_2_O_2_]^2+^ slabs, resulting in the shifts of Bi 4f and Br 3d peaks. The C1s of the samples are shown in Fig. 3c. The peak at 284.6eV is attributed to the contaminated carbon. Besides, two new peak at 286.0 and 288.4 were obtained in BiOBr-OA0.01 sample, which are assigned to sp^2^ hybridized C–O, and C=O bonds, respectively^10^. This indicates the existence of oxalic acid anions on BiOBr-OA0.01 surface.

To further clarify the spatial distributions of oxalic acid anions in the modified BiOBr, XPS depth profiling technique with Ar^+^ sputtering was employed. As shown in Fig. 3d, the peak intensity of carbon–oxygen bonds (at about 288.4 eV) in BiOBr-OA0.01 is decreased during the Ar^+^ sputtering from 0 to 40 s, and vanished at 80 s. This observation suggests that the addition of oxalic acid anions during hydrothermal treatment leads to their existence on the surface of BiOBr, instead of uniform doping in the crystal. As a result, the chelation strategy of surface modification for BiOBr would supply a basis for the generation of SOVs, and would not induce the occurance of bulk oxygen vacancies during the following light irradiation.

**Figure 4:**
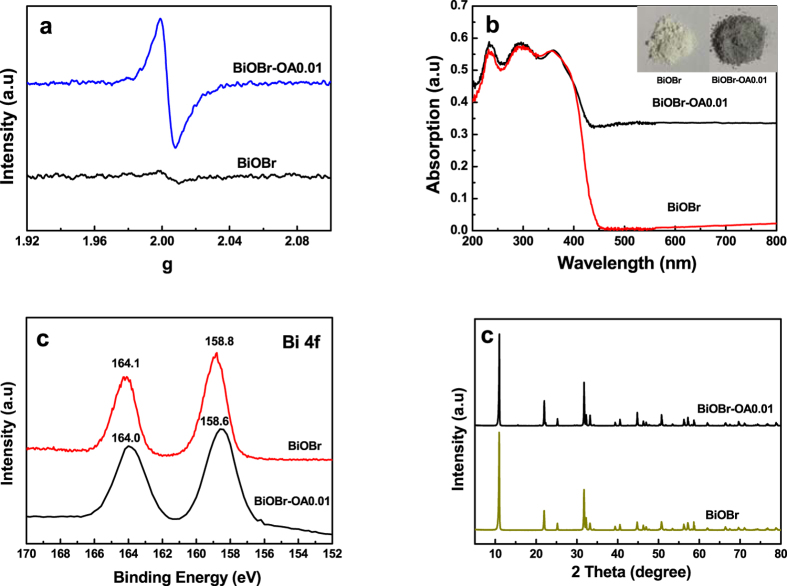
(**a**) ESR spectra, (**b**) UV-vis absorption spectra, (**c**) X-ray photoelectron spectra of Bi 4f, and (**d**) XRD patterns of the as-prepared irradiated BiOBr and BiOBr-OA0.01 photocatalysts.

Based on the strong chelation ability of polybasic carboxylic acids, we design the introduction of oxalic acid for modifying BiOBr aiming at weakening the bond of Bi–O and facilitating the escape of the O atoms on the surface under visible-light irradiation, which acts as a kind of energy. To verify this hypothesis, the structure of irradiated BiOBr and BiOBr-OA0.01 samples were characterized by electron paramagnetic resonance at 77K in liquid N_2_, which is a direct and sensitive method to monitor the presence of oxygen defects. As shown in Fig. 4a, BiOBr-OA0.01 exhibits the apparent peak at g = 2.001, while the irradiated BiOBr does not. This signal has been reported previously, which can be attributed to surface oxygen vacancies^11^,^38^. Therefore, from the ESR result, the SOVs have been possibly formed.

The UV-vis diffuse reflectance spectra of irradiated BiOBr and BiOBr-OA0.01 are shown in Fig. 4b. Compared with BiOBr, the absorption sharp edge of BiOBr-OA0.01 has feeble shift to visible region and the absorbance is enhanced in the range of 450 to 800nm, which is induced by oxygen vacancies. From the inset of Fig. 4b, the color of the as-prepared photocatalysts changes from pale yellow of BiOBr to gray of BiOBr-OA0.01. The Eg of BiOBr is estimated by the following formula^15^:


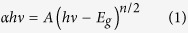


where α, h, ν, A and Eg are the absorption coefficient; Planck constant; the light frequency, a constant; and the band-gap energy, respectively. The value of n is 4 for BiOBr, determined by an indirect transition occurring in the semiconductor. The Eg of BiOBr can be estimated from a plot of (αhυ)^1/2^ versus energy (hυ). The Eg of BiOBr obtained from the tangent intercept is 2.71 eV (as shown in Fig. S1). To further elucidate the electronic structure of BiOBr, electrochemical flat potential measurements were carried out, and the data were plotted in Mott–Schottky coordinates. As shown in Fig. S2, the flat band potentials of BiOBr is roughly −0.93V (*vs.* Ag/AgCl). Accordingly, as a n-type semiconductor, the flat band potentials of BiOBr is estimated to be −0.71 V (pH = 7) after correction of the Ag/AgCl potential of 0.22 V (vs. NHE)^44^. It is generally known that the conduction band (CB) positions of *n*-type semiconductors are about 0.1 V higher than their flat potentials. Thus it is deduced that the CB of BiOBr is about −0.81 V, which was more negative than the standard redox potential of O_2_/•O_2_^−^ (−0.28 V vs NHE)^45^. Combining the Eg of 2.71eV, the relative valence band (VB) position of BiOBr can be inferred at 1.90V.

Figure 4c shows the high-resolution Bi 4f spectra of BiOBr and BiOBr-OA0.01. The binding energies of the Bi 4f_7/2_ and Bi 4f_5/2_ peaks of irradiated BiOBr are located at 158.8 and 164.1eV. In comparison to pure BiOBr, the Bi 4f5_/2_ and Bi 4f_7/2_ peaks of BiOBr-OA0.01 exhibit a shift of about 0.2 eV toward lower binding energy, which is indicative of the presence of lower charge Bi ions in BiOBr-OA0.01^46^,^47^. This result clearly verifies that the Bi^3+^ ions can be reduced to low-charge Bi ions, which is correlated with oxygen vacancies. The XRD pattern of BiOBr-OA0.01 is shown in Fig. 4d. Comparing to bare BiOBr, no other obvious impurity peaks can be detected when oxygen vacancies are introduced. A similar pattern of the adsorption-desorption isotherms for representative BiOBr and BiOBr-OA0.01 were also obtained (as shown in Fig. S3). There are no significant changes in the S_BET_ value (BiOBr: 4.88 m^2^/g, BiOBr-OA0.01: 6.26 m^2^/g).

**Figure 5:**
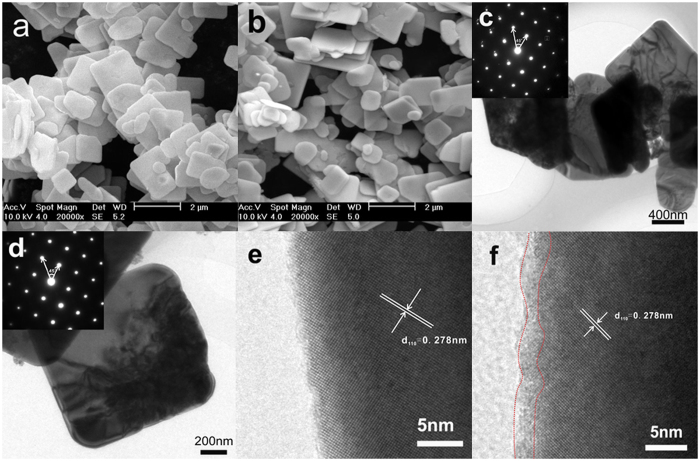
SEM and TEM images (**a**),(**c**),(**e**) of BiOBr and (**b**),(**d**),(**f**) of BiOBr-OA0.01.

The microscopic information of the representative samples were examined by SEM and TEM. As shown from Fig. 5a–d, both the pristine BiOBr and BiOBr-OA0.01 display an aggregated morphology of nanosheets. The {001} facet exposure characteristic of BiOBr and BiOBr-OA0.01 are reflected by the set of diffraction spots in the corresponding fast Fourier transform pattern indexed as the [001] zone axis of tetragonal BiOBr, and further confirmed by the 45^o^ angle between (110) and (200) planes (inset of Fig. 5c,d)^10^,^11^. The high-resolution TEM image revealed the nanosheets are of high crystallinity (Fig. 5e,f). The lattice fringes with an inter-planar lattice spacing of 0.278 nm is indexed as the (110) atomic plane perpendicular to the (001) plane. Furthermore, pristine BiOBr displays perfect lattice features, however the edge of BiOBr-OA0.01 particles becomes dim and disordered, which indicates that the surface structure of BiOBr-OA0.01 is damaged and SOVs are formed^38^.

**Figure 6:**
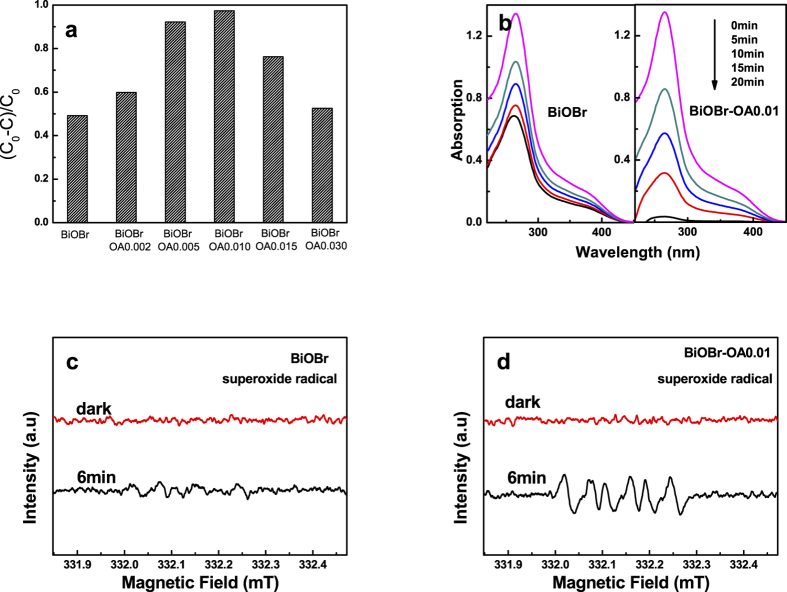
(**a**) Transformation percentage of NBT concentration after 20 min visible light irradiation; (**b**) Transformation process of NBT under visible light irradiation; (**c**,**d**) ESR spectra of DMPO–•O_2_^−^ adducts after visible light irradiation for 6 min over BiOBr and BiOBr-OA0.01.

The photocatalytic activities of pristine irradiated BiOBr and BiOBr-OA samples on enhancing the activation of O_2_ to •O_2_^−^ radicals are fristly investigated by the photocatalytic process of nitro-tetrazolium blue chloride (NBT) under visible light. NBT exhibits an absorption maximum at 265 nm, which can be detected by UV-vis spectrophotometer; however, the product of •O_2_^−^ with NBT does not. Hence, the removal efficiency of NBT can reflect the level of activation on O_2_ to •O_2_^−^ radicals. Figure 6a,b show the transformation percentage of NBT in 20 min irradiation over pristine BiOBr and BiOBr-OA0.01, respectively. It is clear that the photocatalytic activation of O_2_ of BiOBr-OA samples gradually increases till the BiOBr-OA0.01 displays the highest activity. When the concentration of oxalic acid is further increased, the activity begins to decrease. Thus, the activity of modified BiOBr photocatalysts are greatly influenced by the concentration of oxygen vacancies, which can be controlled by the concentration of oxalic acid.

To further directly observe the generation of •O_2_^−^ radicals, ESR technique was employed to monitor the •O_2_^−^ radicals under visible light irradiation. Figure 6c,d show the results of pristine BiOBr and BiOBr-OA0.01 samples employing dimethyl sulphoxide (DMSO) as solvent^48^. It can be seen that the intensity of •O_2_^−^ radicals in BiOBr-OA0.01 suspension is obviously intensive, while the signal in pristine BiOBr is very weak. The result is consistent with the NBT tests. In the BiOBr photocatalytic system, •O_2_^−^ radicals are often considered as the products of photon-generated electron. Thus, this phenomenon indicates that the introduction of SOVs on BiOBr has enhanced the number of effective photon-generated electrons on the CB of BiOBr. Moreover, Xiao *et al*. have demonstrated the molecular oxygen trapped by photo-generated electrons can produce superoxide radicals, which inhibit the recombination of photo-generated charge carries and further improve the photocatalytic activities^43^.

**Figure 7:**
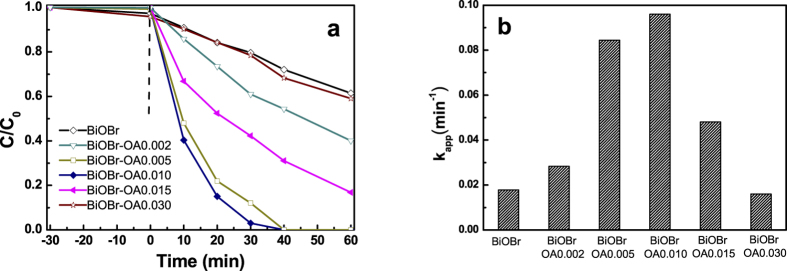
(**a**) Photocatalytic activities and (**b**) the reaction kinetics constant of BiOBr and BiOBr-OA samples on the degradation of MO under visible light irradiation.

The visible light photocatalytic activities of pristine irradiated BiOBr and BiOBr-OA samples were tested by the degradation of methyl orange (MO). In general, the photocatalytic reactions should follow the pseudo-first-order kinetics model when the initial pollutant concentrations are low. The relationship between the illumination time and the concentrations of pollutant can be expressed as follows^15^:


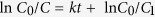


where *k* is the pseudo-first-order rate constant, C_0_ is the original MO (10 mg/L) concentration, C_1_ and C are the concentration after adsorption and at a reaction time *t*, respectively. The photocatalytic activities of pristine BiOBr and BiOBr-OA samples are shown in Fig. 7a,b. Similarly, BiOBr-OA0.01 displays the highest photocatalytic activity. The apparent rate constant *k* over BiOBr-OA0.01 is 0.096 min^−1^, which is about 5.3 times as high as that pristine BiOBr (*k* = 0.018 min^−1^).

In order to test the stability of SOVs, the BiOBr-OA0.01 sample was tested by the repeatability of photocatalytic degradation of MO. As shown in Fig. S4, after eight cycles, there is only a slight loss of activity, due to the loss of photocatalyst. The stability of the SOVs on BiOBr were also examined by EPR and UV-vis absorption spectra. As shown in Fig. S5, the electron spin resonance peak at g = 2.001 is persistent after eight cycles. Besides, the UV-vis diffuse reflectance spectra of the recycled catalyst has not changed (Fig. S6). Thus, the SOVs on BiOBr described in this paper exhibit extraordinary stability.

To further verify the chelation function of carboxylic acid anions on inducing SOVs for BiOBr, many carboxylic acids with different molecular size and structure were selected as the modifier, including formic acid, acetic acid, tartaric acid, etc., which are shown in Table S1. The photocatalytic activity illustrated as rate constant with different additive are shown from Figs S7–S15, respectively. An evident rise of the photocatalytic activity is obtained by adding succinic acid, tartaric acid and citrate acid; however, there was no obvious improvement of the photocatalytic activity by other additive modification. Raman and XPS measurements are employed to detect the crystal structural changes and the surface states by formic acid (FA) anion modification as representative for detecting the difference from oxalic acid. The results are shown in Figs S16 and S17. Compared with pure BiOBr, there is no obvious Raman shift for BiOBr-FA0.01, which illustrates that there is no effective interaction between formate anion and BiOBr. The peak at 288.4eV is also undetected in the XPS high resolution spectrogram of C1s. ESR was employed to further test the oxygen defects of BiOBr-FA0.01. As shown in Fig. S18, no obvious peak can be seen at g = 2.001. These results indicate that there should be no formic acid modified on BiOBr, further supporting the conclusion that oxalic acid anions are existed on BiOBr-OA0.01 surface.

As designed, the generation of SOVs should firstly come from the effective doping of carboxylic acids. The interaction between Bi^3+^ and carboxylic acid anions should play an important role, and the atomic charges of the carboxylic oxygen should be the most critical factor responsible for the surface chelation. Natural bond orbital (NBO) analysis is a well-established tool to predict the atomic charges in molecules. Therefore, we carried out NBO analysis via the procedures contained with Gaussian 03 package. The NBO electrostatic charges of the carboxylic oxygen atom in these carboxylic acid ions are shown in Table 1. From the photocatalytic activity, the NBO values of the carboxylic oxygen listed in Table 1, and the molecular weight and dimension of the additives, it can be concluded that the enhancement of photocatalytic activity is closely related with the NBO values of the carboxylic oxygen, and has no relationship with molecular weight and dimension of the modifiers. Carboxylic acids with higher NBO absolute values, especially greater than 0.830, are effective in modifying BiOBr. This result illustrates that our hypothesis about chelation function of carboxylic acids for metal ions are right, which would be broadened for other metal oxide nanomaterials.

**Figure 8:**
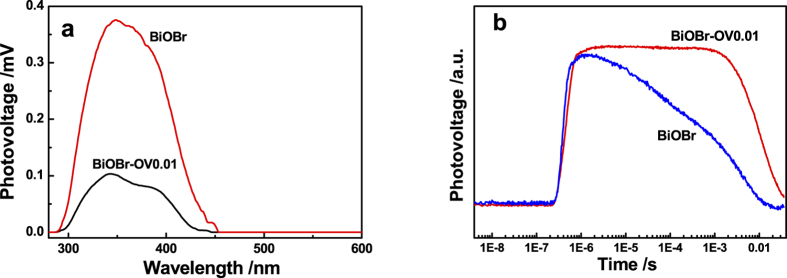
(**a**) The surface photovoltage spectra (SPV) and (**b**) Transient photovoltage (TPV) responses of pristine BiOBr and BiOBr-OA0.01.

Base on the successful introduction of SOVs on BiOBr and their highly oxidation properties, we attempt to clarify the underlying mechanism of the enhancement photocatalytic activity. It is well known that photocatalytic activity is mainly governed by the adsorption ability, crystal phase structure and separation efficiency of photo-generated charges. As mentioned above, there are no obvious changes of the BET surface area and crystal phase structures for BiOBr with and without SOVs. Thus, the separation efficiency of photoinduced charges may play an important role in the photocatalysis. Thus, in order to understand the effect of SOVs, surface photovoltage spectroscopy (SPV) technique is employed firstly to investigate the photo-generated charges transfer properties since a SPV response can be detected^49^ after the separation of photo-generated carriers^50^. Figure 8a shows the SPV spectra of pristine BiOBr and BiOBr-OA0.01. It is seen that there is an evident SPV response areas in the spectrum of BiOBr. The positive photoelectric signal from 300 to 440 nm corresponds to band-to-band transition of BiOBr, which indicates that photo-generated electrons and holes move toward the bulk and surface, respectively^51^. Compared with pristine BiOBr, the SPV signal of BiOBr-OA0.01 in the range of 300–440 nm is decreased significantly. This indicates that the formation of SOVs act as the electron acceptor on the surface, which weakens the effect of surface space-charge region^50^.

Transient photovoltage (TPV) measurement is also used to further investigate the charge dynamics, including the generation, separation, and recombination of the photo-induced electrons and holes^52^. Figure 8b shows the normalized TPV spectrum of pristine and modified BiOBr. Pure BiOBr displays a positive photovoltage transient signal with an abrupt rise at 5 × 10^−7 ^s under the irradiation of a 354 nm laser pulse. Generally speaking, the positive TPV response denotes that the accumulation of positive charges at the top electrode during the irradiation^53^. This phenomenon demonstrates that the drift of positive charge under the self-built electric field at the surface region. Furthermore, the drift velocity of the electrons is much higher than that of holes, so the positive charge in the BiOBr surface can be enriched and detected. Afterward, as a result of recombination, charge concentration on the BiOBr surface decreases which causes a gradual weakeness of the TPV response. The short lifetime indicates a low separation efficiency of the photo-generated charges in pure BiOBr. Similarly to pure BiOBr, the TPV curves of BiOBr-OA0.01 also displays an abrupt rise at 5 × 10^−7^ s, however, compared to pristine BiOBr, the weakened point of the TPV response is from 5 × 10^−7 ^s to 1 × 10^−3 ^s. This phenomenon can actually be considered as the lifetime of the electron in SOVs-modified BiOBr becomes much longer than that of pristine BiOBr. Because the lifetime of the TPV response comes from the recombination of electron-hole pair on the BiOBr surface, the extension of the lifetime indicates that the recombination of electron-hole pairs is greatly suppressed by the introduction of SOVs. Therefore, the improvement of activated O_2_ and photocatalytic activity is mainly resulted from the enhancement of the charge separation efficiency due to the SOVs-induced defect states, which can act as the initial charge carriers acceptor to inhibit electron/hole recombination.

To detect the main active oxidative species during the photocatalytic process, radical scavengers and N_2_ purging were employed. The degradation of MO over BiOBr-OA0.01 under visible light irradiation was choosed as a model reaction. As shown in Fig. S19, there is no evident change for the addition of t-BuOH (6 mM, •OH scavenger). On the contrary, the photocatalytic degradation efficiencies of MO significantly are decreased in the presence of AO (6 mM, h^+^ scavenger)^54^. The results suggest that the holes are the main oxidative species and •OH radicals has no obvious effect on the degradation of MO or there is no •OH radical generated in the photocatalytic process. To illustrate the role of •O_2_^−^ radical, AgNO_3_ as e^–^ scavenger^40^ and N_2_ purging were taken. It is seen that the addition of AgNO_3_ improves the degradation of MO obviously; meanwhile, N_2_ purging decreases the degradation efficiency of MO. The reasons are that the capture of electrons by AgNO_3_ and O_2_ can enhance the separation efficiency of photo-generated carriers, leaving more holes moving to the surface of catalyst to react with MO molecules. This result indicates that •O_2_^−^ is not the main oxidizing species during the degradation of MO; however, the formation of •O_2_^−^ during the photocatalysis process provides benefit to improve the photocatalytic degradation efficiency. Xiao *et al*.^40^ have demonstrated the similar result.

**Figure 9:**
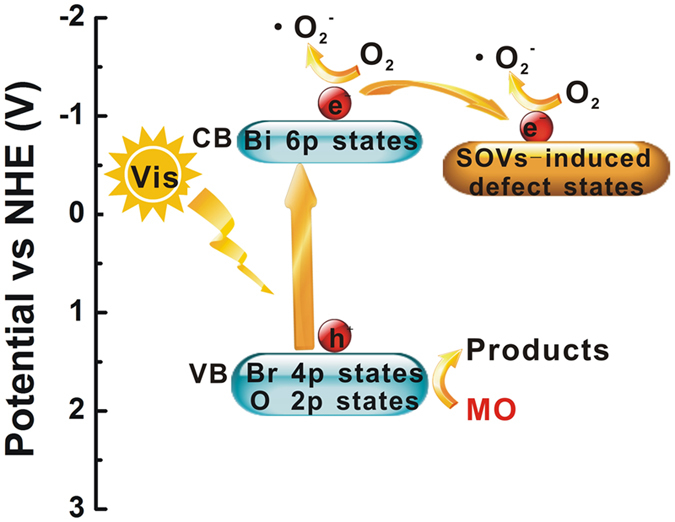
Schematic diagram for the separation of electron-hole pairs and photocatalytic process over the BiOBr with surface oxygen vancancies.

On basis of the above results and analysis, a possible pathway for the charge separation over SOVs-modified BiOBr photocatalyst and the mechanism for the enhanced photocatalytic activity under visible light is proposed as Fig. 9. For pure BiOBr, electrons on the VB of BiOBr are excited to the CB under visible light irradiation. Some electrons are trapped by O_2_ adsorbed on the surface of photocatalyst particles to form •O_2_^−^ radicals. After the introduction of SOVs, the SOVs-induced defect states act as photo-generated electron traps, leaving more holes on the VB of BiOBr. As the electrons trapped on the defects are usually in the metastable state, which can be trapped by O_2_ adsorbed on the surface of photocatalyst to further form •O_2_^−^ radicals, thus the generation of •O_2_^−^ radicals is improved significantly. The enhancement of the •O_2_^−^ radicals can reduce the recombination of photo-generated carrier and improve the separation efficiency of electron-hole. Thus, higher degradability on organic pollutant is attained.

In summary, surface oxygen vancancies are successfully introduced on {001} facets exposed BiOBr nanosheets via a sample surface modification by polybasic carboxylic acids. The introduction of surface oxygen vacancies on BiOBr causes significant enhancement of efficiency of separation of photon-generated carriers, which further show in highly efficiency of activated O_2_ and degradability of methyl orange. The rate constant over BiOBr with surface oxygen vancancies is 5.3 times as high as that pristine BiOBr in degrading methyl orange. The enhanced level of photocatalytic performance is influenced by the concentration and extent of oxygen vacancies, which can be controlled by the concentration of polybasic carboxylic acid anions. The generation of surface oxygen vacancies comes from the effective doping of polybasic carboxylic acids and the subsequent irradiation treatment. Only the polybasic carboxylic acids with NBO absolute values higher than 0.830, such as oxalic acid, tartaric acid, citrate acid and succinic acid can be effective for modifying BiOBr and the surface oxygen vacancies can be obtained. This work would not only provide new insight for activating surface oxygen atoms and fabricating surface defects of nanomaterials, and but also deepen the knowledge of coordination and chelation chemistry in modifying the crystal structure and performance.

## Methods

### Preparation of samples

The carboxylic acids modified BiOBr and pure BiOBr were synthesized by a hydrothermal route. In a typical procedure, 0.5 mmol Bi(NO_3_)_3_·5H_2_O was added to 40 mL oxalic acid solution with different concentrations. The mixture solution was magnetically stirred for 2h and then 0.5 mmol NaBr was added to this solution. After magnetically stirring for 3h, the solution was transferred into a 50 mL Teflon-lined stainless autoclave. The autoclave was heated at 160 °C for 8h. The resulting precipitates were collected, thoroughly washed with deionized water and dried at 80 °C in air. The corresponding samples are designated as BiOBr-OAx, (x is the concentration of oxalic acid solution). Pure BiOBr was prepared according to the same procedure with pure water as the reaction mediator in the absence of carboxylic acid.

The SOVs on BiOBr samples were fabricated by irradiating BiOBr-OA in pure water under a 300 W xenon arc lamp with a 400 nm cutoff filter for 2 hours. The corresponding samples are designated as BiOBr-OAx.

### Characterization

X-ray diffraction (XRD) patterns were measured on a Rigaku D/MAX 2500 X-ray diffractometer equipped with a Cu K_α_ radiation (λ = 0.154 nm) source. Raman measurements were carried out by Thermo DXR Microscope with a 633 nm laser. The Brunauer–Emmett–Teller (BET) specific surface areas (S_BET_) of the samples were analyzed by a Quanta chrome NOVA2000 nitrogen adsorption/desorption apparatus. Surface morphology was observed by scanning electron microscopy (FESEM, HITACHI S-4800). High-resolution transmission electron microscopy (HRTEM) images were collected with a field emission transmission electron microscope (JEOL JEM-2010). UV–Vis diffuse reflectance spectra (DRS) were recorded on a spectrophotometer (Thermofisher Evolution 220). X-ray photoelectron spectroscopy (XPS) measurements were performed on a PHI 1600 ESCA XPS system. All binding energies were calibrated using the contaminant carbon (C 1s = 284.6 eV) as the reference. Electron spin resonance (ESR) measurements were analyzed on a Bruker EMX-8/2.7 X-band ESR spectrometer operating in the X-band at 9.86 GHz and 2.005 mW. The surface photovoltage spectroscopy (SPV) measurements were carried out on a homemade apparatus, which was constituted by a source of monochromatic light with a light chopper, and a lock-in amplifier. The construct of the photovoltaic cell is a sandwich-like structure of ITO-sample-ITO. The transient photovoltage (TPV) signals of the samples were collected with by a 500 MHz digital phosphor oscilloscope (TDS 5054, Tektronix) with a preamplifier. The Mott–Schottky curves performed on an electrochemical workstation (CHI-660E, China) using a standard three electrode system. An aqueous solution containing 0.1 M Na_2_SO_4_ was employed as the electrolyte. The counter and the reference electrodes were platinum wire and Ag/AgCl (saturated KCl), respectively. The as-obtained photocatalyst film electrodes deposited on cleaned 1.5 cm × 1.0 cm fluoride-tin oxide (FTO) glass served as the working electrode.

### Detection of superoxide anion radicals

The amount of superoxide anion radicals (•O_2_^−^) during the photocatalytic process was determined by nitrotetrazolium blue chloride (NBT, 2.5 × 10^−5 ^mol/L). NBT exhibits an absorption maximum at 265 nm, which can be detected by UV-vis spectrophotometer, however, the product of •O_2_^−^ with NBT does not^16^. The test procedures were as follows: Firstly, NBT was dissolved in H_2_O with a concentration of 2.5 × 10^−5 ^mol/L. Then, 0.20 g of the obtained catalyst was dispersed in 200 mL of the NBT solution. Irradiation experiments were carried out under visible light for 20 min, and sampled at an interval of 5min. Finally, the suspension was centrifuged, filtered, and measured on Thermofisher Evolution 220 spectrophotometer.

### Photocatalytic activity evaluation

Methyl orange (MO, anionic dye, 10 mg/L) was selected as the objective pollutants to evaluate the activity of the photocatalysts. 0.20 g of photocatalyst powder was dispersed in 200 mL of dye aqueous solution in a 500 ml photo-catalytic reactor. The suspensions were stirred in darkness at 300 rpm for 30 min in order to reach adsorption equilibrium. Irradiation experiments were carried out under a 300 W xenon arc lamp with a 400 nm cutoff filter. The total light intensity was 0.44 W/cm^2 ^in the range of 400–1064 nm measured by a Newport 842-PE optical power/energy meter. For kinetic studies, samples were taken at regular time intervals and were analyzed by a UV–vis spectrophotometer. To investigate the stability of the as-prepared photocatalyst, the nanopowders were separated from solution and used for the next run experiment.

## Additional Information

**How to cite this article**: Wang, X.-j. *et al*. A Chelation Strategy for *In-situ* Constructing Surface Oxygen Vacancy on {001} Facets Exposed BiOBr Nanosheets. *Sci. Rep.* **6**, 24918; doi: 10.1038/srep24918 (2016).

## Supplementary Material

Supplementary Information

## Figures and Tables

**Table 1:** The NBO values of the carboxylic oxygen.

Additive	NBO (e)	Additive	NBO (e)
oxalic acid	−0.841	succinic acid	−0.834
formic acid	−0.676	tartaric acid	−0.832
acetic acid	−0.797	citric acid	−0.847
propionic acid	−0.798	*n*−pentanoic acid	−0.793
butyric acid	−0.794	adipic acid	−0.818
