# Supplementary material for: A Chelation Strategy for In-situ Constructing Surface Oxygen Vacancy on {001} Facets Exposed BiOBr Nanosheets
Source: Sci Rep. 2016 Apr 26;6:24918. doi: 10.1038/srep24918 (PMC4845065; doi:10.1038/srep24918)
Supplement: Supplementary Information [file srep24918-s1.doc]

**Supporting Information**

A Chelation Strategy for *In-Situ* Constructing Surface Oxygen Vacancy on {001} Facets Exposed BiOBr Nanosheets

Xiao-jing Wang,^1^ Ying Zhao,^2^ Fa-tang Li,^1^ Li-jun Dou,^1^ Yu-pei Li,^1^ Jun Zhao^1^ & Ying-juan Hao^1^

^1^College of Science, Hebei University of Science and Technology, Shijiazhuang 050018, China

^2^College of Science, Agricultural University of Hebei, BaoDing, 071001, China

Correspondence and requests for materials should be addressed to F.T.L. (email: lifatang@126.com)

Table S1. Structure, molecular weight and dimension of the additives

| Additive | Structure | Molecular weight | Dimension(nm)* |
| --- | --- | --- | --- |
| oxalic acid (OA) |  | 90 | 0.532 |
| formic acid (FA) |  | 46 | 0.298 |
| acetic acid (AA) |  | 60 | 0.416 |
| propionic acid (PA) |  | 74 | 0.545 |
| butyric acid (BA) |  | 88 | 0.502 |
| succinic acid (SA) |  | 118 | 0.717 |
| tartaric acid (TA) |  | 150 | 0.621 |
| citric acid (CA) |  | 192 | 0.613 |
| *n*-pentanoic acid(nPA) |  | 102 | 0.602 |
| adipic acid (2AA) |  | 146 | 0.821 |

*The dimension of the additive molecules were calculated using the software of *Chemical Office*

**Figure S1.** Plots of (αhv) ^1/2^ versus photon energy (hv) for the band-gap energy of BiOBr.

**Figure S2.** Mott–Schottky plots of BiOBr electrode in
0.1 mol/L Na_2_SO_4_ at pH=7

**Figure S3.** N_2_ sorption isotherms of the as-prepared BiOBr photocatalysts

**Figure S4.** Recycling tests of a BiOBr-OA0.01 catalyst. Reaction conditions: 0.2 g of catalyst, 200 mL, 10 mg/L MO aqueous solution.

**Figure S5** EPR signals of BiOBr-OA0.01and the catalyst after eight cycles

**Figure S6** UV-vis absorption spectra of BiOBr-OA0.01and the catalyst after eight cycles

**Figure S7.** Reaction kinetics constants (*K_app_)* of methanoic acid modified BiOBr.

FA: formic acid; the value after FA is its molality (in mol·L^-1^)

**Figure S8.** Reaction kinetics constants (*K_app_)* of acetic acid modified BiOBr.

AA: acetic acid; the value after AA is its molality (in mol·L^-1^)

**Figure S9.** Reaction kinetics constants (*K_app_)* of propionic acid modified BiOBr.

PA: propionic acid; the value after PA is its molality (in mol·L^-1^)

**Figure S10.** Reaction kinetics constants (*K_app_)* of butyric acid modified BiOBr.

BA:butyric acid; the value after BA is its molality (in mol·L^-1^)

**Figure S11.** Reaction kinetics constants (*K_app_)* of succinic acid modified BiOBr.

SA: succinic acid; the value after SA is its molality (in mol·L^-1^)

**Figure S12.** Reaction kinetics constants (*K_app_)* of tartaric acid modified BiOBr.

TA: tartaric acid; the value after TA is its molality (in mol·L^-1^)

**Figure S13.** Reaction kinetics constants (*K_app_)* of citric acid modified BiOBr.

CA: citric acid; the value after CA is its molality (in mol·L^-1^)

**Figure S14.** Reaction kinetics constants (*K_app_)* of n-pentanoic acid modified BiOBr.

nPA: n-pentanoic acid; the value after Npa is its molality (in mol·L^-1^)

**Figure S15.** Reaction kinetics constants (*K_app_)* of adipic acid modified BiOBr.

2AA: adipic acid; the value after 2AA is its molality (in mol·L^-1^)

**Figure S16.** Raman spectra of the synthesized BiOBr, BiOBr-OA0.01 and BiOBr-MA0.01 photocatalysts.

**Figure S17.** C1s X-Ray photoelectron spectra of BiOBr and BiOBr-MA0.01

**Figure S18.** ESR spectra of BiOBr and BiOBr-MA0.01.

**Figure S19.** Trapping experiment for detecting active species during the photocatalytic reaction of MO.
